# Electrospun Gelatin/β-TCP Composite Nanofibers Enhance Osteogenic Differentiation of BMSCs and *In Vivo* Bone Formation by Activating Ca^**2+**^-Sensing Receptor Signaling

**DOI:** 10.1155/2015/507154

**Published:** 2015-06-01

**Authors:** Xuehui Zhang, Song Meng, Ying Huang, Mingming Xu, Ying He, Hong Lin, Jianmin Han, Yuan Chai, Yan Wei, Xuliang Deng

**Affiliations:** ^1^Department of Geriatric Dentistry, Peking University School and Hospital of Stomatology, Beijing 100081, China; ^2^Department of Dental Materials, Peking University School and Hospital of Stomatology, Beijing 100081, China; ^3^Department of Prosthodontics, Peking University School and Hospital of Stomatology, Beijing 100081, China; ^4^National Engineering Laboratory for Digital and Material Technology of Stomatology, Beijing 100081, China; ^5^Beijing Laboratory of Biomedical Materials, Peking University School and Hospital of Stomatology, Beijing 100081, China

## Abstract

Calcium phosphate- (CaP-) based composite scaffolds have been used extensively for the bone regeneration in bone tissue engineering. Previously, we developed a biomimetic composite nanofibrous membrane of gelatin/*β*-tricalcium phosphate (TCP) and confirmed their biological activity *in vitro* and bone regeneration *in vivo*. However, how these composite nanofibers promote the osteogenic differentiation of bone marrow mesenchymal stem cells (BMSCs) is unknown. Here, gelatin/*β*-TCP composite nanofibers were fabricated by incorporating 20 wt% *β*-TCP nanoparticles into electrospun gelatin nanofibers. Electron microscopy showed that the composite *β*-TCP nanofibers had a nonwoven structure with a porous network and a rough surface. Spectral analyses confirmed the presence and chemical stability of the *β*-TCP and gelatin components. Compared with pure gelatin nanofibers, gelatin/*β*-TCP composite nanofibers caused increased cell attachment, proliferation, alkaline phosphatase activity, and osteogenic gene expression in rat BMSCs. Interestingly, the expression level of the calcium-sensing receptor (CaSR) was significantly higher on the composite nanofibrous scaffolds than on pure gelatin. For rat calvarial critical sized defects, more extensive osteogenesis and neovascularization occurred in the composite scaffolds group compared with the gelatin group. Thus, gelatin/*β*-TCP composite scaffolds promote osteogenic differentiation of BMSCs *in vitro* and bone regeneration *in vivo* by activating Ca^2+^-sensing receptor signaling.

## 1. Introduction

Calcium phosphate (CaP) ceramic materials have been used traditionally in research into bone regeneration and clinical repair of bone defects because of their favorable biocompatibility and osteoconductivity [1–3]. However, the use of CaP ceramic materials alone is limited because of their brittleness and low plasticity [4]. To overcome these shortcomings, polymer materials have been introduced to form composite scaffolds to improve bone defect repair efficiency and clinical applicability of CaP materials [5–7]. A variety of composite scaffolds combining CaP materials and natural or synthetic polymers have been produced by different preparation technologies. Among them, the electrospinning technique has received increasing attention in regenerative medicine because of its attractive features, such as producing ultrafine fibers that mimic physically the natural bone extracellular matrices (ECM) at the nanoscale [8–10] and the surface morphology, architecture, and performance of these fibers can be modulated by modifying the composition or content of the components [11–13]. Thus, in the field of bone tissue engineering, it is a rational strategy to develop composite scaffolds with nanofibrous structures to recapitalize the extracellular matrix of bone.

In recent years, electrospun CaP/polymer nanofibrous composites have been recognized as beneficial for the attachment, proliferation, and osteogenic differentiation of osteoblasts [14–16], as well as improving the efficiency of bone defect repair [10, 17, 18]. However, the mechanism behind the supportive function of these scaffolds is poorly understood. Recently, Liu et al. reported that nanofibrous hydroxyapatite/chitosan (nHAp/CTS) scaffolds could induce osteogenesis of bone marrow mesenchymal stem cells (BMSCs) through the activation of the bone morphogenetic protein (BMP)/Smad pathway [19]. However, for biodegradable composite materials containing CaP ceramics, understanding how calcium ions released from these nanofibers microenvironment influence the osteogenic differentiation of MSCs* in situ* is of crucial importance for optimizing the design of scaffold materials for bone regeneration applications. Extracellular calcium ions are important to enhance the proliferation and phenotype expression of osteoblast cells [20, 21]. Previous reports showed that the effect of calcium ions on the osteogenic differentiation of osteoblast-like cells MC3T3-E1 [22] or human adipose-derived stem cells [23] is concentration-dependent.

Previously, we successfully prepared gelatin/*β*-TCP composite nanofibers with different contents of *β*-TCP nanoparticles using the electrospinning technique. The results demonstrated that attachment, spreading, proliferation, and differentiation of human osteosarcoma MG-63 cells increased with increasing content of *β*-TCP nanoparticles and continuous release of Ca^2+^ into the medium [24]. In addition, composite nanofibers with a high content of *β*-TCP led to significant bone formation compared with that of the pure electrospun gelatin scaffolds [25]. However, how these composite nanofibers promote the osteogenic differentiation of BMSCs is largely unknown.

The objective of the present work was to analyze the effect of electrospun gelatin/*β*-TCP composite nanofibers on the osteogenic differentiation of rat BMSCs and examine the underlying mechanism* in vitro* and* in vivo*. Initially, we assessed the cell attachment, proliferation, and spreading and alkaline phosphatase (ALP) activity of rat BMSCs on gelatin/*β*-TCP compared with pure gelatin nanofibers. We then detected mRNA levels of osteogenic specific genes and calcium-sensing receptor (CaSR) as a calcium-signaling molecule. Subsequently, we investigated the efficacy of gelatin/*β*-TCP to induce new bone regeneration and related CaSR expression by surgically creating a critical-sized calvarial defects model in rats.

## 2. Materials and Methods

### 2.1. Preparation of Electrospun Nanofibers

**Figure 1:**
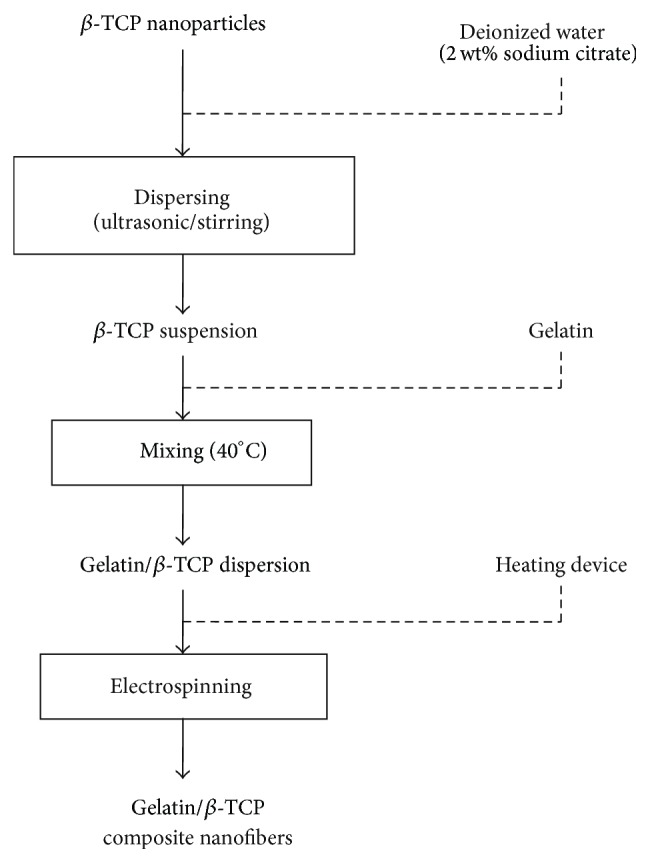
Schematic diagram of the composite nanofibers fabrication process.

The detailed procedure for the electrospinning of gelatin/*β*-TCP solution is shown in Figure 1 and described in our previous work [24]. Firstly, a defined amount of *β*-TCP nanoparticles (average particle size = 200 nm, Rebone Biomaterials Co., Shanghai, China) was dispersed in deionized water containing 2% w/v sodium citrate. Then 20% (w/v) of gelatin (pH 4.5–5.5, Bloom Number 240–270, Amresco, USA) was added into the *β*-TCP suspension solution. The contents of *β*-TCP were set as 20 wt% of the gelatin. Electrospinning was then performed using the following variables: applied voltage 20 kV, solution feeding rate 0.3 mL/h, collecting distance 12 cm, and ambient conditions of 40°C. To prepare scaffolds for cell culture, the electrospun nanofibrous membranes were chemically cross-linked according to our previous research [24]. All electrospun samples were dried for over 3-4 days in a vacuum oven to remove any potential residual solvents.

### 2.2. Characterization of Electrospun Nanofibers

The surface morphology and internal structure of the composite nanofibers were observed using a scanning electron microscope (SEM; Hitachi S-4700, Tokyo, Japan). The distribution of *β*-TCP nanoparticles in the gelatin nanofiber matrix was investigated by transmission electron microscopy (TEM) using a Hitachi H-800 machine. The crystal and chemical structures of the composite nanofibers were examined by X-ray diffraction (XRD; Rigaku D/max 2500 VB2+/PC, Japan) and Fourier transform infrared spectroscopy (FTIR; Nicolet 8700, USA) spectrometry, respectively.

### 2.3. Attachment and Proliferation of rBMSCs

Rat BMSCs (5 × 10^4^ cells/well) were seeded onto experimental scaffolds in 12-well plates and incubated at 37°C in a humidified atmosphere with 5% CO_2_. After 1 day of culture, the samples were fixed in 2.5% glutaraldehyde and serially dehydrated with an increasing ethanol gradient, air-dried in a hood, and sputtered with gold before observation under SEM (S-3000N, Hitachi, Japan). Cytoskeletal organization was observed under a confocal laser scanning microscope (CLSM; FluoView-300, Olympus, Tokyo, Japan). Nuclei were stained with 4′,6-diamidino-2′-phenylindole (DAPI; Vector Laboratories, Burlingame, CA, USA) and actin filaments were stained with rhodamine phalloidin (Molecular Probes, Eugene, OR, USA) after culturing for 24 h. The cell spreading areas were measured using Image J software (National Institutes of Health, Bethesda, MD, USA) employing a random sampling method. Cell proliferation was assayed using a CCK-8 kit (Dojindo, Japan) at 1 day, 3 days, and 7 days of culture, with the absorbance being read at a wavelength of 450 nm, using an enzyme linked immunosorbent assay reader (Bio-Rad, Hercules, CA, USA).

### 2.4. Alkaline Phosphatase (ALP) Activity Assay

Rat BMSCs/scaffolds (*n* = 6) were continually cultured in wells supplemented with osteogenic medium containing 50 mg/mL ascorbic acid-2-phosphate, 100 nM dexamethasone, and 10 mM *β*-glycerolphosphate. At 4, 7, and 14 days, the ALP activity of the adherent cells was assessed using an Alkaline Phosphatase Assay Kit (Abcam, Cambridge, MA), according to the manufacturer's instructions. The absorbance was measured at a wavelength of 405 nm, and values of ALP activity were read off a standard curve based on standard samples provided in the kit.

### 2.5. Quantitative Real-Time PCR Analysis

After osteogenic induction culturing for 7, 14, and 21 days, total RNA was extracted from each sample using the TRIZOL reagent (Gibco-BRL, Gaithersburg, MD, USA), following the manufacturer's instructions. The RNA was then reverse transcribed to generate cDNA using the Reverse Transcription System (Promega, Madison, WI, USA). Real-time RT-PCR was performed using the SYBR Green Detection System with an ABI PRISM 7500 Real-Time PCR System (Applied Biosystems, Foster City, CA, USA). All reactions were carried out in triplicate. The primer sequences of the osteogenic genes, including runt-related transcription factor 2 (*RUNX-2*), collagen type I (*COL1A1*), bone morphogenetic protein-2 (*BMP-2*), osteocalcin (*OCN*), and calcium-sensing receptor (*CaSR*), are listed in Table 1.

### 2.6. Animals and Surgical Procedures

**Figure 2:**
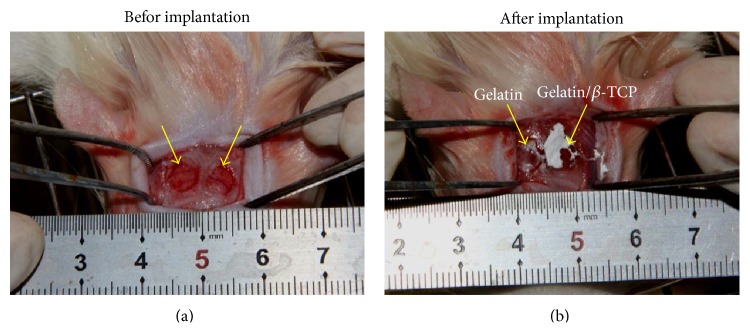
The establishment of rat calvarial defect model. (a) The diameter of the bone defect region was about 5 mm. (b) The nanofibrous scaffold was implanted into the bone defect region. The arrows denote the surgical site.

Twelve 8-week-old male Sprague-Dawley rats were used in this study. The experimental protocol was approved by the Animal Care and Use Committee of Peking University. To establish the calvarial defect model, the rats were anesthetized intraperitoneally with phenobarbitol sodium (100 mg/kg) and the dorsal cranium was exposed. Two critical-sized full thickness bone defects (5 mm diameter) were prepared in each rat at the center of each parietal bone, using a saline-cooled trephine drill (Figure 2). Each defect was flushed with saline to remove bone debris. The left defects were implanted with gelatin/*β*-TCP composite nanofibrous scaffolds and the right defects were implanted with pure gelatin nanofibrous scaffolds as a control. The whole calvarias were harvested for evaluation 4 and 12 weeks after implantation.

### 2.7. Microcomputed Tomography (Micro-CT) Scanning Evaluation

At 4 and 12 weeks after implantation, calvaria samples were harvested intact and fixed in 4% paraformaldehyde for 24 h at 4°C. The specimens were examined using micro-CT scanning, as previously described [26]. Files were reconstructed using a modified Feldkamp algorithm, which was created using microtomographic analysis software (Tomo NT; Skyscan, Belgium). After three-dimensional (3D) visualization, bone morphometric analyses, including calculation of bone mineral density (BMD) and bone volume fraction (Bone volume/total volume, BV/TV), were carried out on the region of interest (ROI).

### 2.8. Histological Analysis

Tissue processing and sectioning were carried out as previously described [26]. Briefly, tissue samples were fixed in 10% neutral buffered formalin for 7 days, decalcified and dehydrated according to standard protocols, embedded in paraffin, and sectioned at 5 *µ*m thickness. Hematoxylin and eosin (H&E) staining and Masson's trichrome staining were performed separately on tissue sections, according to the manufacturer's protocols, and images were captured under a light microscope (CX21, Olympus, Japan).

### 2.9. Immunohistochemical Analysis

Immunohistochemistry for OCN and CaSR was performed as previously described [27, 28]. Briefly, tissue slides were deparaffinized and rehydrated and then submerged in hydrogen peroxide to quench peroxidase activity. Before exposure to the primary antibody against OCN (ab13420, CA 1 : 100, Abcam) and CaSR (ab19347, CA 1 : 100, Abcam), slides were incubated with 1% BSA to block nonspecific binding. After incubation with the primary antibody overnight at 4°C, HRP conjugated secondary antibody was applied to the slides for 1 hour at room temperature. Finally, a diaminobenzidine (DAB; Beyotime, Jiangsu, China) kit was used to develop the color, followed by counterstaining with hematoxylin. Slides were observed under a light microscope (CX21, Olympus, Japan). OCN and CaSR expression within the defect area was quantified using a web application ImunoRatio [29].

### 2.10. Statistical Analysis

All quantitative data were expressed as means ± standard deviation (SD). Statistical analyses were performed using the SPSS 19.0 software (Chicago, IL). Statistical differences were determined using Student's* t*-test for independent samples. Differences between groups of ^*^ *P* < 0.05 were considered statistically significant and ^**^ *P* < 0.01 was considered highly significant.

## 3. Results and Discussion

### 3.1. Characteristics of Electrospun Gelatin/*β*-TCP Composite Nanofibers

**Figure 3:**
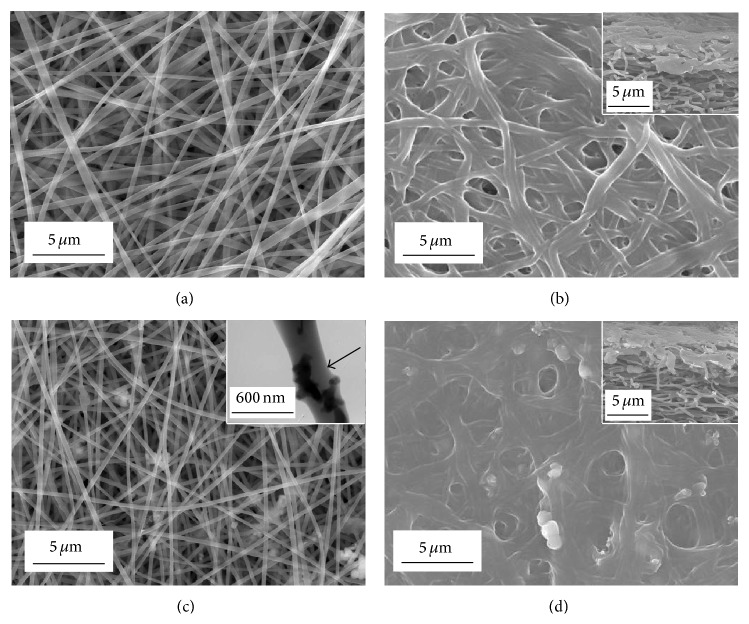
SEM images of electrospun nanofibers before ((a) and (c)) and after cross-linking ((b) and (d)). ((a) and (b)) Gelatin nanofibers; ((c) and (d)) gelatin/*β*-TCP composite nanofibers. The insets in (b) and (d) show the sections of cross-linked gelatin nanofibers and gelatin/*β*-TCP composite nanofibers, respectively. The TEM image of a gelatin/*β*-TCP composite nanofibers is denoted by a black arrow in (c).

Figure 1 shows the morphology of electrospun gelatin and gelatin/*β*-TCP composite nanofibers. All the electrospun nanofibers showed a nonwoven structure, with an interconnected porous network. Pure gelatin nanofibers were continuous, smooth, and homogeneous (Figure 3(a)). Composite nanofibers had a rough surface because of the incorporation of *β*-TCP nanoparticles (Figure 3(c)). It has been reported that a rough nanofiber surface created by apatite particles could promote cell adhesion, proliferation, and osteogenic differentiation of bone-forming cells [30]. It could be inferred that the *β*-TCP nanoparticles were embedded in the nanofibers, which was confirmed by the TEM image (the inset of Figure 3(c)). Gelatin is water-soluble, so gelatin/*β*-TCP composite nanofibers must be cross-linked before being subjected to cell culture. To illustrate the cross-linking effect of the nanofibers, we observed the surface and side of the nanofibrous scaffolds. The nanofibers were curled and conglutinated with each other throughout the scaffolds (the insets of Figures 3(b) and 3(d)) after being cross-linked. The diameter of the fibers increased clearly because the fibers swelled during the cross-linking treatment, while the pore size decreased significantly in comparison with the noncross-linked samples.

**Figure 4:**
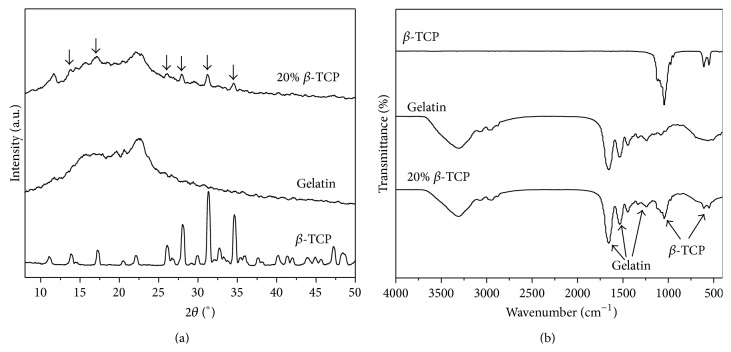
XRD (a) and FT-IR (b) patterns of gelatin/*β*-TCP composite nanofibers.

Figure 4(a) shows the XRD pattern of composite nanofibers. The diffraction peaks of *β*-TCP could be observed in the gelatin/*β*-TCP composite nanofibers. Meanwhile, the structure of gelatin was not affected by the incorporation of *β*-TCP and the electospinning process. Their presence and chemical stability was further confirmed by the FT-IR spectra shown in Figure 4(b). The absorption bands corresponding to both gelatin (amide group: ~1650, 1550, and 1250 cm^−1^) and *β*-TCP (PO_4_^3−^: 950–1100 and 550–620 cm^−1^) were detected clearly. In our previous study, electrospun gelatin/*β*-TCP composite nanofibers with 20 wt% *β*-TCP possessed remarkable effects in terms of the bioactivity of osteoblasts-like MG-63 cells* in vitro* [24] and guided bone regeneration* in vivo* [25]. Therefore, in this study, gelatin/*β*-TCP composite nanofibers with 20 wt% *β*-TCP were employed to further investigate that how the process of osteogenic differentiation of BMSCs and bone defects repair* in situ* was promoted by these composite nanofibers.

### 3.2. Composite Nanofibers Enhanced* In Vitro* Bioactivity of rBMSCs

**Figure 5:**
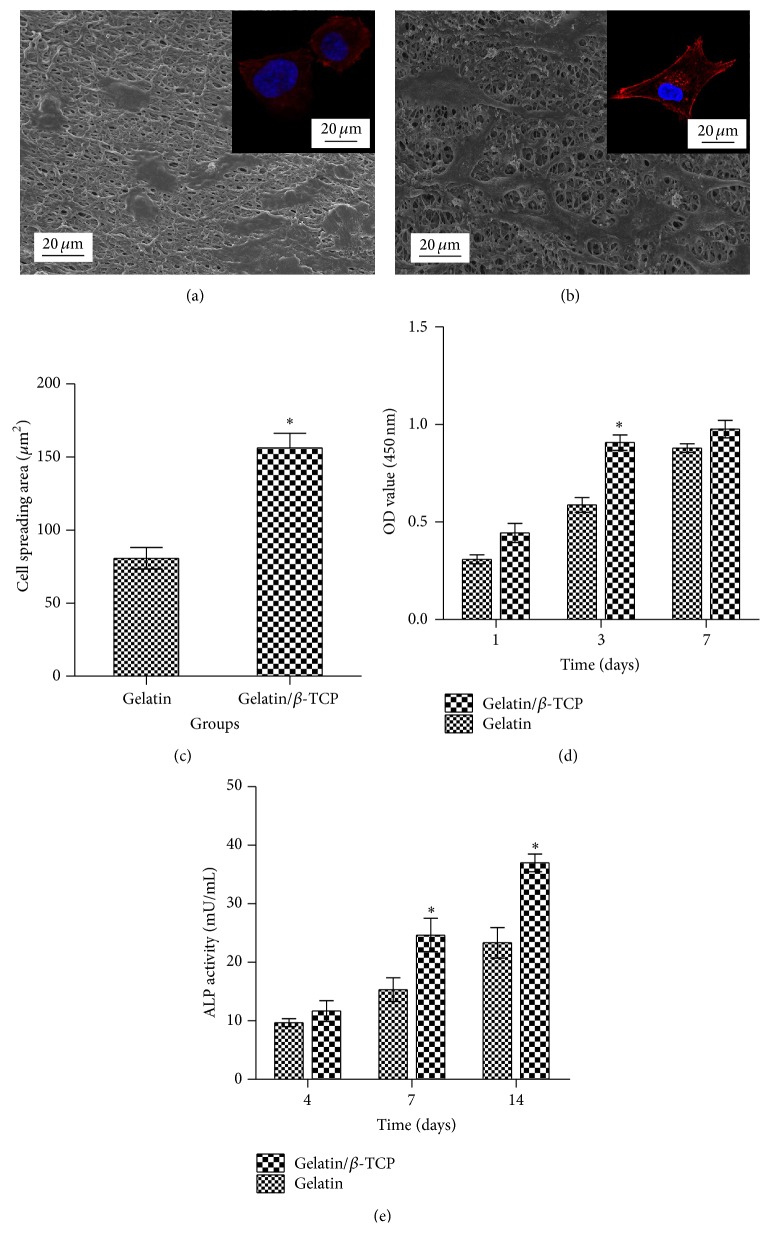
*In vitro* bioactivity of rBMSCs on nanofibrous scaffolds. ((a) and (b)) SEM images of rBMSCs seeded on (a) gelatin and (b) gelatin/*β*-TCP composite scaffolds after 24 h of culture. Insets show representative images of the cytoskeleton. (c) Proliferation of rBMSCs grown on various scaffolds as assessed by a CCK-8 assay. (d) The measured cell spreading areas. (e) Alkaline phosphatase (ALP) activity of rBMSCs cultured on various scaffolds at 4, 7, and 14 days.

Figures 5(a) and 5(b) show the SEM and CLSM images of rBMSCs after seeding on the cross-linked gelatin and gelatin/*β*-TCP nanofibrous scaffolds for 24 h. Generally, cells attached onto the scaffolds displayed a flat and well-spread morphology, and the actin filaments were organized in well-defined stress fibers throughout the cells. Interestingly, rBMSCs seeded on composite nanofibrous exhibited more apparent cellular processes (Figure 5(b) and the inset), as well as a larger cell spreading area (Figure 5(c)) compared with cells grown on pure gelatin nanofibers. This was largely related to the increased surface roughness caused by the incorporation of *β*-TCP nanoparticles and subsequently enhanced protein absorption ability, as confirmed by our previous research [24]. The cell proliferation rate on the composite nanofibrous scaffolds was also higher than that of pure gelatin (Figure 5(d)). However, cell proliferation rate became decreased on the composite scaffolds and showed no significant difference compared to that of the pure gelatin group on the 7th day. This slight proliferation suppressive effect is possibly related to the differentiation tendency of rBMSCs, because there is a reciprocal relationship between cell proliferation and differentiation [31].

To evaluate the effect of nanofibers on the early osteogenic differentiation ability of rBMSCs, ALP activities were quantified at days 4, 7, and 14 after cell seeding. As shown in Figure 5(e), higher ALP activity was observed on the composite nanofibrous scaffolds compared with that of pure gelatin nanofibers. This may be explained by the sustained release of calcium ions from biodegradable *β*-TCP, as reported by our previous study [24] and other studies [32, 33]. These results suggested that electrospun gelatin/*β*-TCP composite nanofibers encouraged enhanced attachment, well-organized cytoskeleton, improved proliferation, and high ALP activity of rBMSCs* in vitro*. Our results are in line with previous studies demonstrating that electrospun poly(L-lactic acid) (PLA)/20% TCP accelerates osteogenic differentiation of human adipose-derived stem cell compared with neat electrospun PLA scaffolds [33]. Similarly, Lü et al. reported that the introduction of hydroxyapatite (HA) into electrospun poly(3-hydroxybutyrate-co-3-hydroxyvalerate) (PHBV) nanofibers could induce MSCs to differentiate into osteoblasts [18].

### 3.3. Composite Nanofibers Upregulated Osteogenesis-Related Gene Expression and Activated Calcium-Sensing Receptor Signaling

**Figure 6:**
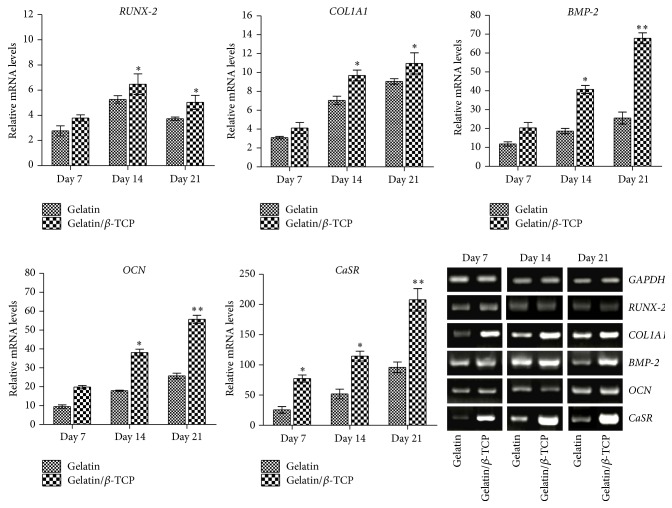
The mRNA expression levels and gel panels of the RT-PCR products of osteogenic genes and CaSR in rBMSCs cultured on electrospun nanofibrous scaffolds. The relative expression levels are normalized to the reference gene* GAPDH* and relative to TCPs in the basic medium.

In our previous study, the Ca^2+^ release behavior from composite nanofibers with different *β*-TCP contents in cell culture medium was estimated by refreshing the medium every 2 days. The results showed that the concentration of Ca^2+^ increased with the content of *β*-TCP loading and the highest Ca^2+^ concentration was reached in 20 wt% *β*-TCP loading [24]. In this work, we further investigated that how the osteogenic differentiation of BMSCs was promoted by Ca^2+^ released from these composite nanofibers. The expression levels of osteogenic genes of rBMSCs on nanofibrous scaffolds were evaluated in the osteogenic induction culture, as shown in Figure 6. The transcript levels of* RUNX-2*,* COL1A1*,* BMP-2,* and* OCN* on gelatin/*β*-TCP composite scaffolds were higher than those on pure gelatin nanofibers. This promotion effect could be ascribed to the Ca^2+^ released from composite nanofibers.

To examine the relation between released Ca^2+^ and osteogenic differentiation of BMSCs, we examined the expression of* CaSR* in rBMSCs. Interestingly, the expression of* CaSR* in the composite materials group was significantly higher than that of pure gelatin group. Agarose gel electrophoresis of the PCR products showed a similar trend in the quantitative data. This result implied that CaSR may contribute to osteogenic differentiation of BMSCs mediated by composite scaffolds containing *β*-TCP. CaSR is reported to act as a sensor, thus transducing the Ca^2+^ signaling to intracellular gene expression to regulate cell function [34], and has been studied extensively* in vitro* and* in vivo* [28, 35–37]. However, Barradas et al. suggested that CaSR is not involved in mediating BMP-2 expression of MSCs in different concentration of Ca^2+^ medium [38]. This may be ascribed to the difference of stimulation approach of Ca^2+^. In our work, the activation of CaSR in promoting osteogenic differentiation of rBMSCs may be a comprehensive effect regulated by both the structural property of the nanofibers and the sustained Ca^2+^ release.

### 3.4. Composite Nanofibers Promoted* In Vivo* Bone Formation

**Figure 7:**
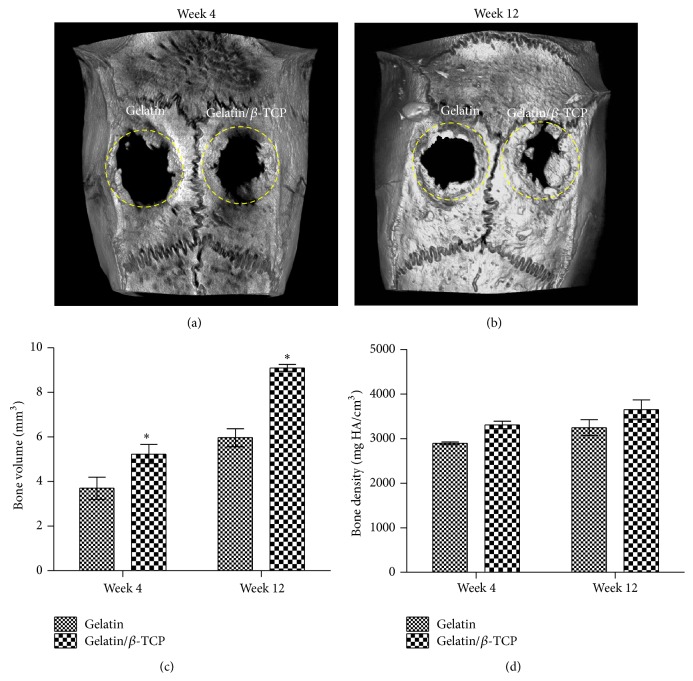
Micro-CT analysis of rat calvarial defects repair. ((a) and (b)) Representative 3D *µ*-CT images of rat calvarial defects at 4 weeks (a) and 12 weeks (b) after implantation. ((c) and (d)) The quantitative analysis of the bone volume and bone density at 4 weeks (c) and 12 weeks (d) after implantation. Dashed circles denote the bone defect regions. (^*^ *P* < 0.05).

To investigate the guided bone regeneration ability of electrospun gelatin/*β*-TCP nanofibrous scaffolds and confirm the activation of CaSR signaling* in vivo*, the calvarial defect in rat was chosen as the experimental animal model because it is a common model and has been adopted widely by many researchers [10, 19, 39, 40]. In this work, two circular (5 mm diameter), full thickness critical defects were made in the cranium of each rat. Our main goal was to investigate whether the composite scaffolds had a better guided bone-regeneration capacity than the pure gelatin. Therefore, we treated the left defect with gelatin/*β*-TCP composite scaffolds and used the right defect implanted with pure gelatin scaffolds as a control.  Figure 7 shows the micro-CT analysis results of rat calvarial defects repair at 4 and 12 weeks after implantation. Based on the 3D images, nascent bone formation occurred from the outer margin to the central region in both groups during the implantation process. More bone in-growth could be observed in the composite scaffolds group compared with the pure gelatin group. The whole defect was almost repaired by bone-like tissue at 12 weeks in the composite scaffolds group (Figure 7(b)). Quantification of the bone volume in the defect showed that the gelatin/*β*-TCP composite scaffolds group was significantly elevated compared with the pure gelatin group (*P* < 0.05) at 4 and 12 weeks (Figures 7(c) and 7(d)). The bone density at the two time points was higher in the gelatin/*β*-TCP group compared with that in pure gelatin group but not significantly.

**Figure 8:**
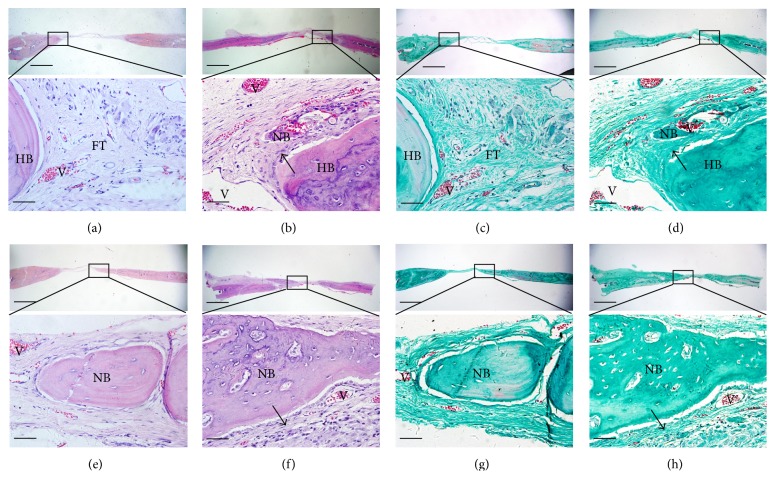
Histological analysis of bone formation at ((a)–(d)) 4 weeks and ((e)–(h)) 12 weeks after implantation. ((a), (b), (e), and (f)) H&E staining; ((c), (d), (g), and (h)) Masson's trichrome staining; ((a), (c), (e), and (g)) gelatin group; ((b), (d), (f), and (h)) gelatin/*β*-TCP group. Black arrows denote the regularly patterned fibrous tissues. (HB: host bone; NB: nascent bone; V: vessels; FT: fibrous tissue). Scale bar = 800 *µ*m (low magnification) and 75 *µ*m (high magnification).

**Figure 9:**
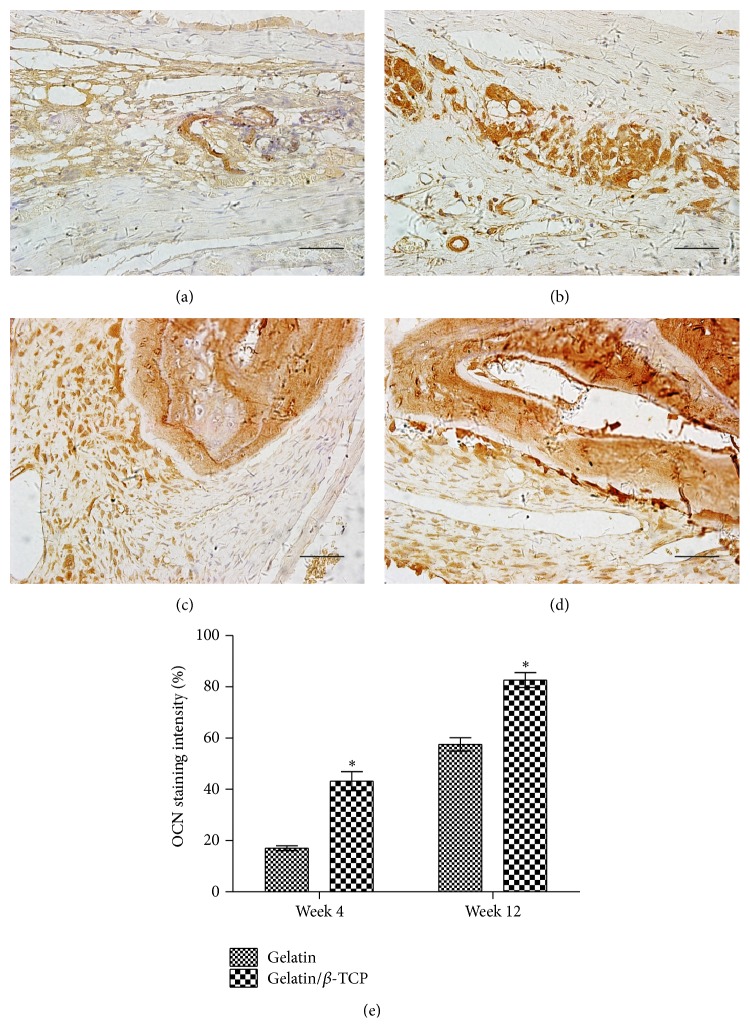
OCN production in rat calvarial defects at 4 and 12 weeks after implantation. ((a)–(d)) Immunohistological staining of OCN after implantation with gelatin ((a) and (c)) and gelatin/*β*-TCP ((b) and (d)) for 4 weeks ((a) and (b)) and 12 weeks ((c) and (d)). Scale bar = 75 *µ*m. (e) The quantitative analysis of the staining intensity of OCN. (^*^ *P* < 0.05).

The newly formed tissues within the calvarium defect were further analyzed by histological staining, as shown in Figure 8. After implantation for 4 weeks, fibrous tissues were formed adjacent to the original bone nodules in the pure gelatin group (Figure 8(a)). Masson staining showed that the fibrous tissue mainly comprised newly formed collagen fibers (Figure 8(c)). In the composite scaffolds group, H&E staining revealed obvious bone structures and abundant vascularization in the middle of the bone defect region (Figure 8(b)), while Masson staining showed more regularly aligned collagen fibers that filled the bone defect region (Figure 8(d)). After implantation for 12 weeks, in the gelatin group, H&E staining revealed the formation of mature bone structures integrating into the bone defect region (Figure 8(e)). In the composite scaffolds, H&E staining revealed that significantly increased bone mass had formed to fill the defect region (Figure 8(f)). Immunohistochemical analysis showed that the region implanted with gelatin/*β*-TCP composite scaffolds expressed a higher level of OCN than the pure gelatin groups at 4 and 12 weeks after implantation (Figure 9). Thus, the quantitative data supported the histological observation (Figure 9(e)). Collectively, these results indicated that electrospun gelatin/*β*-TCP composite nanofibers have a positive effect in guiding bone regeneration. The present results were consistent with another research [10] and our recent report [25]. However, it has also been reported that gelatin/*β*-TCP sponges did not significantly improve bone formation compared with pure gelatin [41]. This discrepancy may reflect differences in the structure properties of scaffold materials and their clinical applicability in different bone defect sites.

### 3.5. Composite Nanofibers Enhanced CaSR Expression* In Vivo*

**Figure 10:**
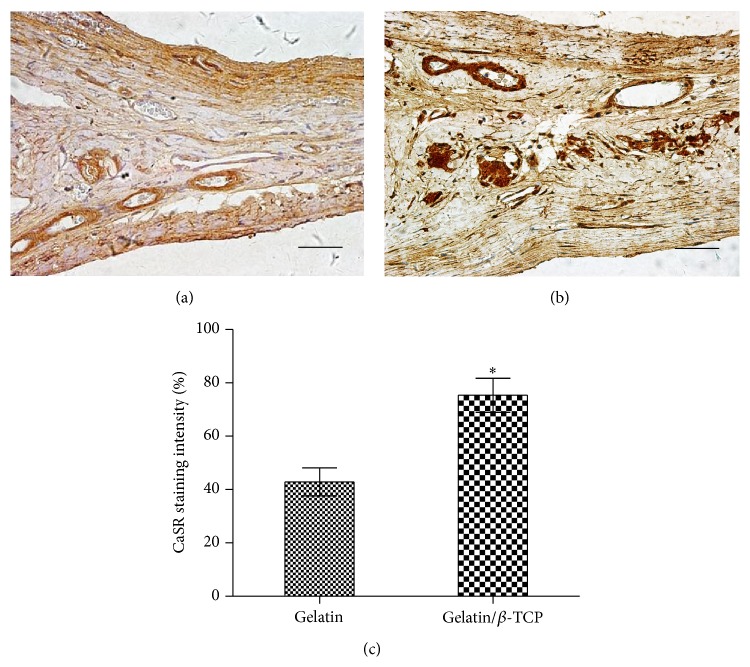
CaSR production in rat calvarial defects at 12 weeks after implantation. ((a) and (b)) Immunohistological staining of CaSR after implantation with gelatin (a) and gelatin/*β*-TCP (b). Scale bar = 75 *µ*m. (c) The quantitative analysis of the staining intensity of CaSR. (^*^ *P* < 0.05).

To assess the effect of implantation with gelatin/*β*-TCP composite nanofibrous scaffolds on CaSR expression, we examined the expression of CaSR in bone regeneration region after 12 weeks of implantation. As shown in Figure 10, more intense staining was observed in the gelatin/*β*-TCP group (Figure 10(a)) compared with the pure gelatin group (Figure 10(b)) and the quantitative analysis results supported this tendency (Figure 10(c)). These results suggested that gelatin/*β*-TCP composite scaffolds promote bone regeneration* in situ* by activating Ca^2+^-sensing receptor signaling.

## 4. Conclusions

In the present study, the nanofibrous gelatin/*β*-TCP composite scaffolds, which have compositional and structural features close to natural bone ECM, supported rBMSCs adhesion, spreading, and proliferation and ALP activity. Furthermore, gelatin/*β*-TCP composite scaffolds induced osteogenic differentiation of BMSC* in vitro* by activating Ca^2+^-sensing receptor signaling. Finally, the gelatin/*β*-TCP composite exhibited more extensive osteogenesis and higher CaSR expression* in vivo *compared with pure gelatin nanofibers. This study highlighted the great potential of the gelatin/*β*-TCP composite nanofibers in the practical application in orthopedics and dentistry, such as guided bone regeneration membranes in periodontal pockets.

## Figures and Tables

**Table 1:** Primer sequences used for real time RT-PCR.

Target gene	Forward primer sequence (5′-3′)	Reverse primer sequence (5′-3′)
*RUNX-2 *	GAGATTTGTAGGCCGGAGCG	CCCTAAATCACTGAGGCGGT
*COL1A1 *	TGGTTTCCCTGGTGCTGC	GGGACCAACTTCACCAGGAC
*BMP-2 *	TGCTCAGCTTCCATCACGAAG	TCTGGAGCTCTGCAGATGTGA
*OCN *	GACCCTCTCTCTGCTCACTCTG	GCTCCAAGTCCATTGTTGAGG
*CaSR *	TTCGGCATCAGCTTTGTG	TGAAGATGATTTCGTCTTCC
*GAPDH *	GGTCGGTGTGAACGGATTTGG	GCCGTGGGTAGAGTCATACTGGAAC
